# Psychometric properties of the ethical conflict in nursing questionnaire critical care version among Chinese nurses: a cross-sectional study

**DOI:** 10.1186/s12912-021-00651-x

**Published:** 2021-07-28

**Authors:** Yuanfei Liu, Nianqi Cui, Yuping Zhang, Xiyi Wang, Hui Zhang, Dandan Chen, Shunxia Sun, Jingfen Jin

**Affiliations:** 1 The Second Affiliated Hospital Zhejiang University School of Medicine (SAHZU), No.88 Jiefang road, Shangcheng District, Hangzhou, 310009 Zhejiang Province China; 2 School of Nursing, Shanghai Jiao Tong University, Shanghai, China; 3 Faculty of Nursing, Zhejiang University School of Medicine, Hangzhou, Zhejiang China; 4 Department of Nursing, The General Hospital of Chongqing, Chongqing, China; 5 Changxing Branch Hospital of SAHZU, No.66 Taihu middle road, Changxing Country, Huzhou, 313100 Zhejiang China

**Keywords:** Ethical conflict, Chinese version, COSMIN checklist, Psychometric properties, Reliability, Validity

## Abstract

**Background:**

Ethical conflicts are common in the critical care setting, and have compromised job satisfaction and nursing care quality. Using reliable and valid instruments to measure the ethical conflict is essential. This study aimed to translate the Ethical Conflict in Nursing Questionnaire — Critical Care Version into Chinese and determine the reliability and validity in the population of Chinese nurses.

**Methods:**

Researchers obtained permission and followed the translation-backward method to develop the Chinese version of the Ethical Conflict in Nursing Questionnaire — Critical Care Version (ECNQ-CCV-C). Relevant psychometric properties were selected according to the Consensus-based standards for the selection of health status measurement instruments checklist. Critical care nurses were recruited from two tertiary public hospitals in Hangzhou, Zhejiang Province, and Kunming, Yunnan Province. Of the 264 nurses we approached, 248 gave their consent and completed the study.

**Results:**

The ECNQ-CCV-C achieved Cronbach’s alphas 0.902 and McDonald’s omega coefficient 0.903. The test-retest reliability was satisfactory within a 2-week interval (intraclass correlation coefficient = 0.757). A unidimensional structure of the ECNQ-CCV-C was determined. Confirmatory factor analysis supported acceptable structure validity. Concurrent validity was confirmed by a moderate relation with a measure for hospital ethical climate (*r* = − 0.33, *p* < 0.01). The model structure was invariant across different gender groups, with no floor/ceiling effect.

**Conclusions:**

The ECNQ-CCV-C demonstrated acceptable reliability and validity among Chinese nurses and had great clinical utility in critical care nursing.

**Supplementary Information:**

The online version contains supplementary material available at 10.1186/s12912-021-00651-x.

## Background

Ethical conflict is referred to as a problem that arises when personal ethical values are not compatible with the organizational values. In the specific field of health care, it features the stress involved in ethical decision-making [1]. The intensive care unit (ICU) is a complex and highly stressful workplace with intensive and often demanding workload. Critical care nurses are at higher risk of confronting end-of-life decisions, physical restraints and futile treatments; to make matters worse, they often have low control and autonomy in daily clinical practice [2–4]. As a consequence, ethical conflict is pervasive in numerous nursing scenarios. Examples include taking care of a patient who should in a general ward rather than an intensive care unit, implementing a treatment that is too aggressive for the patient and causes additional suffering, or making the best use of available techniques and resources for critically ill patients without significantly improving their outcomes [4, 5]. These experiences can lead to subsequent deleterious effects both personally and organizationally. On an individual level, ethical conflicts bring barriers to decision-making. Critical care nurses who experience ethical conflicts would be involved in depression, anxiety, anger, powerlessness and even emotional exhaustion combined with physical symptoms [6]. These experiences render them more prone to burnout, compassion fatigue, job dissatisfaction, and thus leaving the profession of nursing [7, 8]. On an organizational level, a high turnover rate would compromise the quality of nursing care, and poor staffing pattern would in turn aggravate the experience of ethical conflict [9].

In previous studies, the ethical conflict comprises several different types. The most frequently used term is moral distress which was initially defined by Andrew Jameton in 1984. It is referred to as a situation in which a person was constrained from acting upon what he knows to be ethically appropriate [10]. The highlight of this concept is that a decision has been made according to what one considers right. Another type of ethical conflict is moral dilemma, the meaning of which is similar to that of moral distress. It occurs when one has to choose between equally ethically appropriate decisions. Jameton also identified moral uncertainty as a type of ethical conflict in which nurses feel ambiguous if there are ethical problems or recognize that there are problems, but don’t know what the ethical principles are [10]. In 1989 Judith Wilkinson proposed moral outrage to describe the feeling of powerless in the face of other people’s immoral behaviors [11]. Later, Falcó-Pegueroles used moral wellbeing (the coherence of moral thoughts and actions) and moral indifference (the dearth of interest and position towards ethical issues) to depict the absence of ethical conflict [12].

In fact, ethical conflict is a complex construct that involves different moral states. An understanding of ethical theories can be helpful in being aware of ethical issues and defining the source of conflicts. Principle Theory proposed by philosopher Ross is a model of ethics in which four key constructs— autonomy, beneficence, non-maleficence and justice — were used to guide one’s moral action [13]. This theory projects a systematic view of ethical conflict faced by ICU nurses. To be specific, respect for autonomy pertains to the problem of informed consent, beneficence provokes discussion in the balance between patients’ interests and available resources, observance of confidentiality and protection of privacy are important consideration of non-maleficence, and the principle of justice implies equal access to healthcare [13–15]. Critical care scenarios are embodied in the four constructs which provide an explicit theoretical basis that has been widely used in terms of ethical issues. Nursing scenarios going against these constructs can trigger ethical conflicts [14]. Therefore, it is necessary to measure these constructs of ethical conflict faced by ICU nurses as well as identify different conflict areas and explore the root causes of ethical conflicts.

Considering the severity of ethical conflict in clinical practice, several instruments were developed for quantitatively measuring ethical conflicts. Corley initially developed the Moral Distress Scale (MDS) in the guide of Jameton’s conceptualization of moral distress, House and Rizzo’s role conflict theory, and Rokeach’s theory on values and value systems. The MDS comprises 32 items using Likert 7-points to measure the level of moral distress [16]. Several years later, Hamric formed the MDS-Revision (MDS-R) by shortening and updating scale items. The MDS-R consists of 21 items in a 4-point Likert format scoring the frequency and intensity of moral distress. To enhance the applicability of the MDS-R, Hamric adapted six parallel versions focusing on adult and pediatric nurses, physicians and other healthcare providers, but it does not include ICU setting [17]. Another tool called the Moral Distress Thermometer uses visual analogue and 0–10 rating scales to describe how much moral distress one has been experiencing, but the utility of this rapid screening tool need to be test [18].

The existing instruments have only centered on the constructs of moral distress which is only a part of ethical conflict. Thus, these scales may be insufficiently extensive to evaluate the ethical conflict faced by ICU nurses. The sole use of frequency and intensity of moral distress is not adequate to explain the essence of ethical conflicts in critical care scenarios [12]. It is essential to learn about the variable “exposure to ethical conflict” which is the product of frequency and intensity of ethical conflict and analyze the relation between the conflict types and exposure to conflict. This would identify the barriers that block the ethical decision-making more precisely [19]. Furthermore, based on a thorough review of the literature, there were no studies that jointly examined the types of ethical conflict depicted by Jameton and Wilkinson (moral uncertainty, moral dilemma, moral distress and moral outrage) [10, 11] and considered the state of absence of ethical conflict (moral indifferent and moral wellbeing) [12].

Fortunately, the Ethical Conflict in Nursing Questionnaire-Critical Care Version (ECNQ-CCV) brings a new perspective on analyzing ethical conflict. It was developed by Falcó-Pegueroles in 2013 and compromised 19 critical care nursing scenarios. In addition to moral uncertainty, moral dilemma, moral distress and moral outrage, ECNQ-CCV embraced another two states of absence of ethical conflict — moral indifference and moral wellbeing. The range from moral indifference to moral outrage represents a continuum of the presence–absence of ethical conflict. Overall, ECNQ-CCV describes four variables concerning the ethical conflict: frequency, intensity, exposure to the conflict (which is the product of the former two variables) and the types of ethical conflict [12]. Since the moral residue based on the crescendo effect is a common feeling lingering after repeated ethically problematic situations, it is significantly essential to measure the exposure to ethical conflict in ICU setting by the score of frequency multiplied by intensity [20, 21]. Therefore, the ECNQ-CCV is a sensitive tool to detect the exposure to ethical conflict and discriminate different types of conflicts.

To date, the ECNQ-CCV was adapted into the Portuguese [22] and Persian [23] versions and tested to be reliable and valid. Both the original and modified versions have been applied among diverse populations in several countries, including Spain [24], Portage [22], Iran [25] and the United States [26]. Falcó-Pegueroles also conducted a further study regarding the association between the level of exposure and types of the ethical conflict. The range from moral indifference to moral outrage is in an ascending order, which helps explicate the phenomenon, design strategies to mitigate ethical conflicts, and improve the nursing work environment [19].

Since ethical conflicts among ICU nurses are a never-ending problem all over the world, it is significant to develop a universal instrument so that discussions can be held across borders. Although the cultural background of China is different from that of Spain where ECNQ-CCV was developed, there are a lot of similarities in the conflict areas in ICU setting around the world. Based on a literature review, we found that Spanish nurses reported higher exposure to conflict in the situation of ineffective analgesia and lack of engagement in clinical decision-making [24], while futile treatment, end-of-life care and poor communication were the typical conflicts in China [27, 28]. Ethical conflicts appear to arise from specific aspects of nursing care (e.g. resource management) and organizational constrains. Furthermore, ethical conflicts have a lot in common under different cultural background [29]. Therefore, we assumed that Chinese ICU nurses are as susceptible to ethical conflicts as those from many other countries — they in practice face similar conflicts, which are consistent with what ECNQ-CCV can capture.

ECNQ-CCV has been proved to work as a reliable and effective tool to identify different sources of ethical conflicts among ICU nurses in many other countries. However, evidence on psychometric properties of the ECNQ-CCV in the context of Chinese culture remains unknown. When introducing instruments from different cultural background and languages, it is important to translate linguistically accurately as well as to ensure cultural appropriateness to maintain the construct [30]. Therefore, we used COSMIN (COnsensus-based Standards for the selection of health status Measurement INstruments) checklist [30, 31] as a guideline to test the validity (content validity, structural validity, cross-cultural validity, and Criterion validity), reliability (internal consistency, split-half reliability and test-retest reliability), and floor/ceiling effect among critical care nurses and used the STROBE (Strengthening the Reporting of Observational studies in Epidemiology) statement to report the study [32].

## Methods

### Study design and participants

A cross-sectional, descriptive study was conducted in China from October 2020 to March 2021. Two hundred sixty-four critical care nurses from Hangzhou, Zhejiang Province, Kunming, Yunnan Province were recruited according to the convenience sampling. The departments covered a wide variety of nursing critical care units, including the Emergency Intensive Care Unit (EICU), Surgical Intensive Care Unit (SICU), Cardiac Surgical Intensive Care Unit (CSICU), Neurosurgery Intensive Care Unit (NICU) and General Intensive Care Unit for a representative data. A provincial male nurse association also granted permission to conduct the study for a balance between genders in the sample.

The inclusion criteria were participants who (a) were registered nurses and had critical care working experience for at least half a year; (b) were able to give informed consent. The exclusion criterion was to be a nursing student or have an intern in ICU. The online questionnaire was sent to the participants personally using a web-based hospital system. We made sure that they understood each item of the ECNQ-CCV before the formal study. The online questionnaire required an answer for every question, so that there could not be any missing data. We guaranteed all the participants the confidentiality of their private information and their right to withdraw from the study at any stage.

### Instruments

#### Demographic characteristic

An investigator-developed form comprising 10 questions was used to collect socio-demographic information about the participants, including age, sex, education level, religion, years in critical care units, job title, and training in nursing ethics. This form was attached to the ECNQ-CCV-C.

#### Ethical conflict in nursing questionnaire-critical care version (ECNQ-CCV)

Falcó-Pegueroles developed the ECNQ-CCV consisting of 19 scenarios related to critical care nursing in 2013. Each scenario includes three questions measuring the frequency, intensity and types of the ethical conflict. The frequency and intensity are rated on a six-point (0–5) and five-point (1–5) Likert scales, respectively. The percentage is used to report the type of conflict. The level of exposure to the ethical conflict is acquired through multiplying the frequency by its intensity. The score of each scenario ranges from 0 to 25 and the total score is 25 × 19 = 475. Higher scores indicate a greater exposure level of the ethical conflict [12]. Low (< 1 standard deviation (SD) below the mean), moderate (±1 SD around the mean) and high (> 1 SD above the mean) exposure to the ethical conflict were defined [24]. The initial Psychometric evaluation of the ECNQ-CCV showed great internal consistency (Cronbach’s α = 0.882), content validity, and construct validity. The Exploratory Factor Analysis indicated a unidimensional structure [12].

#### Hospital ethical climate survey (HECS)

The 26-item HECS developed by Olson in 1995 is a five-point (1–5) Likert scale to assess the ethical climate in hospitals. It has five dimensions: the relationships between nurses and nurses, patients, doctors, administers and organization. Higher scores added by each item represent a better ethical climate in hospitals [33]. As reported, the Cronbach’s α coefficient of the Chinese version of the HECS is 0.91 [34]. The HECS was used to test concurrent validity.

#### Professional quality of life scale (ProQOL)

The ProQOL developed by Stamm comprises 30 items with five-point (1–5) Likert scale to measure the professional quality of life in the domain of compassion satisfaction, burnout, and secondary traumatic stress [35, 36]. The cut-off score of the three dimensions were < 37, > 27 and > 17, respectively. In the adapted Chinese version of the ProQOL, the Cronbach’s α coefficients of each subscale were 0.87, 0.73 and 0.84 [37]. The ProQOL was used for testing predictive validity.

### Developing the Chinese version of the ECNQ-CCV

We had access to the original English version of the ECNQ-CCV and obtained permission from Falcó-Pegueroles to use and translate the scale into Chinese. Based on the guideline for the process of cross-cultural adaptation [38], the translation stages were as followed:

- Stage I: Two translators who excelled at English and Chinese translated the original ECNQ-CCV into Chinese independently. One translator was a nursing postgraduate familiar with the context in the scale, while the other translator didn’t have a medical background.
- Stage II: After discussion and resolving any discrepancies, the two translators reached an agreement on a synthesis version.
- Stage III: Two native English speakers with Chinese competence, who were completely blind to the original ECNQ-CCV and had no medical background, translated the synthesis version back into English and created two back-translations.
- Stage IV: An expert committee consisting of an ethicist, a methodologist, a language professional, an Emergency and critical care specialist and translators reviewed all reports. The original developer was consulted for confirmation about any question of item meanings. The expert panel then reached consensus on discrepancies and ambiguities, and produced a pre-final version that achieved semantic, idiomatic, and conceptual equivalence.
- Stage V: The pilot study was conducted in 32 critical care nurses according to the guideline which recommended a sample size of 30–40 participants for pre-testing [38]. After completion, the participants were encouraged to provide suggestions and comments on the revision to make expressions clearer. With minor modification, the pre-final version of ECNQ-CCV showed great comprehensibility and legibility.
- Stage VI: The final English version was sent to Falcó-Pegueroles for confirmation. Compared with the original one, there were no differences. Thus, the Chinese version of the ECNQ-CCV (ECNQ-CCV-C) was generated for evaluating the psychometric properties. The original and Chinese versions of the ECNQ-CCV are attached in Supplementary material.

### Data analysis

All data analyses were performed using SPSS Version 25.0 (SPSS Inc.), AMOS version 24.0 (IBM Corp.) and jamovi version 1.6.15 (jamovi project, 2021). Two-tailed tests were calculated with a *P*-value of 0.05 as the significance level. Descriptive analyses were used to report the mean and standard deviation (SD) or medians (25th percentile, 75th percentile) of the continuous variables and the percentage frequency of the categorical variables. We investigated the normality of the distribution of continuous variables using the Kolmogorov-Smirnov test. According to the COSMIN checklist [30, 31], we tested the content validity, structure validity, concurrent validity, predictive validity, internal consistency, test-retest reliability, and a floor/ceiling effect of the ECNQ-CCV-C.

#### Content validity

Content validity is to describe the degree to which the content of an instrument adequately reflect the construct to be measured [39]. Under the guideline of COSMIN checklist, content validity was evaluated by an expert panel based on the methodological criteria for good content validity [40]. Six experts were consulted including a nursing ethics expert, an ICU nursing specialist, an ICU physician, an advanced nursing practitioner, a nursing researcher and a nursing professor. The rating system consists of 10 questions: five for relevance, one for comprehensiveness, and four for comprehensibility. The evaluation was graded as +, − or?, depending on if there was ≥85% of the items meet the criteria (+) or < 85% (−) or insufficient information, respectively [41].

#### Structure validity

Structure validity is an indicator whether the scores of an instrument adequately reflect the dimensionality of the construct to be measured [39]. Maximum likelihood confirmatory factor analysis (CFA) was performed to evaluate the structural validity of the ECNQ-CCV-C. Our sample size in this study met the requirement of recommendation for CFA (200 is considered adequate) [42]. A non-significant chi-square Index (χ^2^) is desirable. However, when in large sample size, the χ^2^ is often significant, and standardized root mean square residual (SRMR) and root mean square error of approximation (RMSEA) are more suitable to assess the goodness of fit [43, 44]. Therefore we relied on the following standards to evaluate model fit: χ^2^ /degrees of freedom ratio (CMIN/DF) < 3.0 [45], SRMR < 0.08, RMSEA < 0.08, goodness-of-fit index (GFI) > 0.90, comparative fit index (CFI) > 0.90 [46, 47]. Also, GFI and CFI above 0.85 and RMSEA below 0.10 were judged to be acceptable as marginal fit [48]. Besides, modification indices (MIs) were inspected to improve the fit of the model.

#### Cross-cultural validity

Cross-cultural validity is required to evaluate measurement invariance when a scale will be used in different “cultural” groups, e.g., different demographic groups (language, age, gender or ethnic) and different population groups [39]. We test the cross-cultural validity of the ECNQ-CCV-C in relation to gender (male vs. female) using multi-group CFA to assess whether factor structure and model fit are acceptable across different gender groups. The sample sizes of each group meet the criteria of more than 5 times the number of items and ≥ 100 [49].

#### Criterion validity

Concurrent validity indicates the degree of correlation between an instrument and a gold standard [39]. Since there isn’t a gold standard in the field of nursing ethics, the relation between the ECNQ-CCV-C scores and the total/subscale scores of HECS were calculated. To test predictive validity, we examine the correlation between the ECNQ-CCV-C and the ProQOL. Both the concurrent validity and predictive validity were analyzed by the Spearman correlation coefficient. The correlation was low as *r* = 0.00–0.30; moderate as *r* = 0.31–0.60; high as *r* > 0.60 with a *P*-value of 0.05 as the significance level [50].

#### Internal consistency

Internal consistency refers to the degree of interrelation among the items [39]. Apart from Cronbach’s alpha, McDonald’s omega coefficient, which was more accurate to explore the internal consistency, was also calculated [51, 52]. We used the Spearman-Brown coefficient and Guttman split-half coefficient for the split-half reliability test to evaluate the internal consistency of the ECNQ-CCV-C. Sufficient homogeneity of the items is based on a score above 0.70. Item total correlation was also calculated to determine the correlation between each item in the scale and the studied construct. Values above 0.4 were desirable [53].

#### Test-retest reliability

Test-retest reliability is defined as to what extent the scores from repeated measurements don’t change over time [39]. To describe the test-retest reliability of the ECNQ-CCV-C, 30 critical care nurses were selected to complete the scale after a 14-day interval. We chosen this time interval between test and retest because it is long enough to prevent the recall of previous answers, though short enough for the condition to change in most cases [54]. Intraclass correlation coefficients (ICC) with a two-way mixed-effects model and an absolute agreement definition were calculated based on the acceptable level ranging from 0.75 to 0.90 [55, 56].

#### Measurement error

Measurement error represents the changes in the scores that are not attributed to true changes in the construct of measurement [39]. Standard Error of Measurement (SEM) was analyzed according to the formula: SEM = SD ×$\sqrt{1-\mathrm{ICC}}$. The standard deviation (SD) from scores comes from all data at the initial assessment. The smallest detectable change (SDC) was then calculated using the formula: SDC = SEM × 1.96 × $\sqrt{2}$ / $\sqrt{\mathrm{n}}$ [57, 58].

#### Interpretability

Interpretability was assessed by floor/ceiling effect. Less than 15% of nurses achieving the highest or lowest score in the ECNQ-CCV-C were deemed as no floor and ceiling effects [39].

## Result

### Participant characteristics

Of the 264 nurses, 248 completed questionnaires for analysis, with a response rate of 94%. Nurses who were 26–30 years (39.9%), female (52.0%), non-religious (98.8%), and had Baccalaureate (78.2%), worked in critical care nursing for 1–5 years (44.8%) count the most. 72.2% of the nurses had acquired knowledge based in ethics and 31.5% nurses had to take on extra workload except for clinical nursing care. The mean score of the ECNQ-CCV-C was 103.94. Further details are presented in Table 1.

**Table 1:** Participant characteristics (*n* = 248)

variable	n (%) or mean ± standard deviation
Age (years)	
18–25	63 (25.4)
26–30	99 (39.9)
31–40	77 (31.1)
41–50	9 (3.6)
Gender	
Men	119 (48.0)
Women	129 (52.0)
Education level	
College or lower	8 (3.2)
Baccalaureate	194 (78.2)
Certificate	39 (15.7)
Master or above	7 (2.8)
Religion	
Yes	3 (1.2)
No	245 (98.8)
Range of clinical experience in critical care (years)	
0.5–1	41 (16.5)
1–5	111 (44.8)
6–10	59 (23.8)
11–15	24 (9.7)
16–20	8 (3.2)
> 20	4 (2.0)
Nursing ethics training experience	
Yes	179 (72.2)
No	69 (27.8)
Clinical workload expect for nursing care	
Only nursing care	170 (68.5)
Nursing management	70 (28.2)
Nursing education	109 (44.0)
Nursing research	56 (22.6)
ECNQ-CCV-C	103.94 ± 56.59
HECS	103.25 ± 12.62
Associates	16.50 ± 2.21
Doctors	21.81 ± 3.81
Patients	16.00 ± 2.16
Organization	23.87 ± 3.03
Administers	25.07 ± 3.46
ProQOL	91.64 ± 12.78
Compassion satisfaction	29.67 ± 6.68
Burnout	27.73 ± 3.22
Secondary traumatic stress	34.23 ± 4.99

*ECNQ-CCV* Ethical Conflict in Nursing Questionnaire-Critical Care Version, *HECS* Hospital Ethical Climate Survey, *ProQOL* Professional Quality of Life Scale

**Table 2:** Correlations of the ECNQ-CCV-C and HECS subscales

	HECS total scale	Nurses	Patients	Doctors	Administers	Organization
ECNQ-CCV-C total scale	−0.33^**^	−.032^**^	− 0.27^**^	−0.22^**^	−.031^**^	− 0.25^**^

**P < 0.01

**Table 3:** Correlations of the ECNQ-CCV-C and ProQOL subscales

	ProQOL total scale	Compassion satisfaction	Burnout	Secondary traumatic stress
ECNQ-CCV-C total scale	0.16^*^	0.18^**^	0.33^**^	−0.04

**P < 0.01; *P < 0.05

### Reliability

The ECNQ-CCV-C presented excellent internal consistency with Cronbach’s alpha (α) being 0.902 and McDonald’s omega (ω) being 0.903. The α and ω value if the item is eliminated ranged from 0.893 to 0.900 and 0.895 to 0.902, respectively. There was no sign of growth both in α and ω coefficient by deleting any item. As for split-half reliability, the Spearman-Brown coefficient was 0.925, and the Guttman split-half coefficient was 0.920. Item total correlation also demonstrated high reliability ranging from 0.410 to 0.664. In the test-retest analysis, the ICCs for the ECNQ-CCV-C were 0.757 (0.280–0.918, 95% CI). The SEM was 34.99, and the SDC indicating the smallest individual change was 6.16.

### Content validity

An acceptable content validity was ensured by the expert panel. The ECNQ-CCV-C was appraised as sufficient (more than 85% of the items meet the criteria of evaluation) for its relevance and comprehensibility. As for comprehensiveness, two experts consider the concept “moral indifference” somewhat negative. After explanation, the experts showed agreement that there were no difficulties in understanding all items. According to experts’ views, the ECNQ-CCV-C reflected the construct of ethical conflict and was feasible to measure the ethical conflict among Chinese critical care nurses populations.

### Structure validity

A series of preliminary data indicated that the ECNQ-CCV-C items were suitable for factor analysis (Kaiser-Meyer-Olkin index = 0.871 and Bartlett’s test of sphericity *p* < 0.001) and demonstrated a unidimensional structure with all the items loading on a single major factor [12]. Our results of CFA were consistent with the original findings. The initial model indices suggested a poor fit based on the RMSEA, GFI and CFI. [χ^2^ = 438.752, df = 152 (*p* < 0.05), CMIN/DF = 2.887, SRMR = 0.066, RMSEA = 0.087(0.078, 0.097 90%CI), GFI = 0.841, CFI = 0.822]. The modification indices showed potential misfits within the questionnaire, as the two items that asked about the relationship between staff were related to each other (item 9 “Working with medical staff who I consider to be professionally incompetent.” and item 12 “Working with a nurse or nursing assistant who I consider to be professionally incompetent.”). By examining the Model fit statistics through pairing items 9 and 12, it indicated an improvement in all the indices and achieved a better acceptable fit [χ^2^ = 397.857, df = 151 (*p* < 0.05), CMIN/DF = 2.635 < 3.0, SRMR = 0.064, RMSEA = 0.081(0.072, 0.091 90%CI), GFI = 0.852, CFI = 0.847]. The standardized factor loading of the item for the ECNQ-CCV-C were all significant (*p* < 0.001) and ranged between 0.425 to 0.704.

### Cross-cultural validity

Multiple-group CFA was conducted to test if the construct was being measured the same way across the demographic variables of gender (women vs. men). An unconstrained model fit for the indices [χ^2^ = 618.450, df = 302 (p < 0.05), CMIN/DF = 2.048, SRMR = 0.088; RMSEA = 0.065 (0.058, 0.073; 90% CI)] suggested the acceptability of an integral goodness-of-fit for different gender subgroups, demonstrating no gender differences. Therefore, the construct in the ECNQ-CCV-C is valid for both male and female nurses.

### Criterion validity

For concurrent validity, the ECNQ-CCV-C demonstrated a moderate and negative correlation of *r* = 0.33 with the HECS, ranging from 0.22 to 0.32 on the subscales, which respectively measure the relationships between nurses and nurses (*r* = 0.32), patients (*r* = 0.27), doctors (*r* = 0.22), administers (*r* = 0.31) and organization (*r* = 0.25) (*p* < 0.01 for all). For predictive validity, the ECNQ-CCV-C had a positive correlation of *r* = 0.33 with burnout measured by ProQOL (*p* < 0.01). A low correlation was found between the average score of ECNQ-CCV-C and the score of compassion satisfaction (*r* = 0.18, *p* < 0.01). There was no significant correlation between the ECNQ-CCV-C and secondary traumatic stress subscale (*p* > 0.05). Further details are presented in Tables 2 and 3.

### Floor/ceiling effect

No critical care nurses (0%) achieved the lowest (0) and the highest (475) scores, demonstrating the absence of floor/ceiling effect.

## Discussion

In this study, we have successfully translated the ECNQ-CCV into Chinese. The translation process was undertaken strictly according to the methodology guideline [38] to ensure the equivalence of content and structure. Under the COSMIN checklist, the reliability and validity of the ECNQ-CCV-C were also examined by applying the scale in a sample of critical care nurses. The results indicated acceptable validity, satisfactory reliability, and no floor/ceiling effect of the ECNQ-CCV-C. Only 10–15 min are required to complete the scale. We propose that the ECNQ-CCV-C is an appropriate tool for assessing the ethical conflict among critical care nurses in mainland China.

Ethical conflicts have received increasing interest in the field of critical care. The mean score of exposure to the ethical conflict was 103.94 (SD = 56.59, range 19–295), which is slightly below the findings in the Spanish [12] and Portuguese [22] samples. Low, moderate and high exposure to the ethical conflict were referred to as < 47.35, 47.35–160.53 and > 160.53, respectively. 73.4% critical care nurses in our sample had low and moderate exposure to the ethical conflict. Although we have fully informed the nurses about confidentiality, due to the resilience and implicitness embedded in Chinese people’s characteristics, it is possible that some critical care nurses reported overly optimistic for their self-rated exposure to ethical conflict [59, 60]. The most challenging situation that ethical conflicts arise from is when a nurse is compelled to provide treatment considered futile and the analgesic pain management he/she takes is ineffective. The result is in line with previous studies [12, 22, 23]. Increasingly, studies concerning nursing ethics and critical care nursing have also focused on these conflicts in the common ICU context.

Despite the one-factor structure, the mean scores vary according to different conflict areas and nurses’ exposures to conflict differ greatly among the constructs of theory. We found that nursing scenarios related to withholding and withdrawing treatments and resource management were noteworthy sources of ethical conflict. From the Principle Theory’s perspective, these high level of conflicts can be explained by the key principle of beneficence [13]. Nurses probably feel overwhelmed by weighing the benefits of patients’ interests against the cost of available resources. They are supposed to not only take the ethical responsibility to relieve the suffering of patients, but also fully consider the patients, family and even lawsuit especially when telling the truth [61, 62]. On the other hand, ethical conflicts arise from clinical practice going against non-maleficence principle (e.g. confidentiality) were less reported in our study. It seems that more nurses attach importance to protecting patients’ clinical data and privacy. While another possible explanation is that nurses are unaware of the problem of sharing information with medical staff who are not directly involved in the patient’s care. They probably don’t regard this situation as an ethical problem. Ethical self-awareness is pressingly needed to some extent. Despite the complexity of nursing clinical practice, ECNQ-CCV-C can work as an effective instrument to explore the essence of ethical conflicts based on the Principle Theory. It is theoretically plausible that ECNQ-CCV-C can capture the concepts of ethical conflict within China.

Based on the study findings, the ECNQ-CCV-C had acceptable internal consistency, which corresponded with the findings of three earlier studies reported in Spain [12], Portugal [22] and Iran [23], suggesting that the sound reliability of ECNQ-CCV-C was supported among Chinese population as well. It indicated that the scores could be repeated under several conditions and free from measurement error to a large degree. The Cronbach’s α of the ECNQ-CCV-C is 0.902, which is above that of the original Spanish version and the Portuguese version, but slightly below the value of the Persian version. A moderate item-total score correlation (*r* = 0.410–0.664) implied favorable internal homogeneity for the ECNQ-CCV-C. The ICCs of the ECNQ-CCV-C, which was absent in the sample of Spanish and Portuguese nurses added another source of evidence to support the reliability of the scale, demonstrating the stability of the ECNQ-CCV-C over time. In addition, a dearth of the ceiling/floor effect for the ECNQ-CCV-C total score also support its applicability in Chinese critical care nurses.

A unidimensional structure of the ECNQ-CCV-C was confirmed by CFA, though the performance of model fit indices was less satisfactory when compared with the original instrument. This is similar to the structural validity reported in previous study of Iranian nurses [23]. And it is difficult to infer the applicability in the Portuguese version because the study gave little detail about factor analysis and did not employ CFA [22]. At first, the one-factor model of the ECNQ-CCV-C has been debatable because of the disparate findings. However, due to the fact that a few scenarios in some factors lack compatibility with each other, the unidimensional structure of the ECNQ-CCV-C was still favored. Moreover, the explanation is supported by the finding of some scholars who viewed ethical conflict as a kind of umbrella concept that captures some entangled moral attributes, such as compromised integrity, interior suffering, detachment from personal values and beliefs, conflicting feelings, powerlessness, etc. These sub-concepts are difficult to discrete from each other, and they are recommended to be delineated altogether [62]. Hence, we implemented the incorporation of modification indices to improve the goodness of fit. The two items that asked about nurses’ relationship with medical staff and nursing assistant had potential and acceptable correlation. Although a significant χ^2^ value was found in our model, previous researches showed that it is probably sensitive to sample size and is prone to be statistically significant when the sample size is large (more than 250) and the number of variables is high (more than 12) [41, 43]. The compromise in model fit could be related to the meaning of language in the Chinese context, but the factor loadings in the range of 0.425 to 0.704, SRMR and RMSEA suggested satisfactory validity for the ECNQ-CCV-C in our sample. We also included a number of male participants to achieve a balanced gender proportion and enlarged the generalizability of the scale to all nurses. No cross-gender differences were observed regarding the ECNQ-CCV-C in the male and female nurse subgroups. This result provided evidence in support of its cross-culture validity.

Consistent with previous studies, we found a significant correlation between the ECNQ-CCV-C and the HECS, suggesting that ethical conflict may be associated with the ethical climate in hospitals. Critical care nurses who worked in an ethically supportive environment may experience lower level of ethical conflict. This indicated that better collaboration and communication in the working environment may help diminish ethical conflict triggers [63–65]. We also expected that the lower exposure to the ethical conflict could result in higher compassion satisfaction of nurses measured by ProQOL. However, the correlation between compassion satisfaction and ethical conflict was low, albeit statistically significant, which was similar to the prior study [66]. When facing ethical conflict, nurses may also find a sense of achievement and personal happiness in their work of helping others, it probably has a positive impact on their compassion satisfaction. Burnout of critical care nurses in this study was positively correlated with the experience of ethical conflict. Higher exposure to ethical conflict was a predictor of higher burnout corresponding well with the previous researches. However, the outcome of the non-significant relationship between the ECNQ-CCV-C and secondary traumatic stress subscale was contrary to the prior findings [67, 68]. Secondary traumatic stress was referred to as work-related secondary exposure to people who have suffered from extreme events [35]. The low risk of secondary traumatic stress among Chinese nurses may be attributed to Chinese people’s cultural differences and contextual characteristics, which may act as a buffer against these stressors.

The ECNQ-CCV-C has several potential clinical implications in healthcare aiming to alleviate ethical conflicts among ICU nurses. First, on the individual level, the assessment of ethical conflict from a self-evaluated perspective may raise nurses’ awareness of this problem. Second, it can also highlight the need for intervention and help nursing administrators implement pragmatic strategies to tackle ethical conflicts in daily practice, such as optimizing the rules and regulations on the organizational level. Third, on the global level, a universal instrument would probably facilitate the discussions about ethical conflicts to be held across border [69]. However, despite that almost all nurses indicated to have experienced ethical conflicts in clinical environment, a few of them showed the need for help to rate the scale because of the difficulty in understanding the concepts of different moral states. Therefore, a comprehensive training session and instructive support during the assessment process is necessary.

There are also some limitations in our study. Firstly, although our sample size suffices the statistical analyses, according to the rules of thumb regarding sample size for CFA, two to three times the amount of 10 participants per variable was recommended [63]. Thus, several more nurses would be favorable. Secondly, the nurses we had access to are almost Han ethnic group, non-religious and only from two provinces in China. Since the ethical conflict they experienced might differ from other populations, our findings may not represent the opinions of all nurses in China, leading to less explanatory power in ethnic minority and religious groups. Thirdly, the cross-sectional study design may have an influence on the assessment of the predictive validity for some adverse outcomes. Also, it does not favor the follow-ups to detect sensitive changes over time in the studied construct, nor can we assess the responsiveness of the ECNQ-CCV-C. Longitudinal representative population-based studies are needed for further validation. To our knowledge, this is the first study to evaluate the psychometric properties of the ECNQ-CCV in China. It is possible that the factor structure emerged in the study is influenced by the norm and social culture of the sample. Future researchers could employ appropriate adjustments to the ECNQ-CCV-C and retest the items in the application to verify the validity of its model. The validation of the ECNQ-CCV-C into different cultural settings will enhance the generalizability of this scale.

## Conclusion

The findings of the cross-section study confirmed that the Chinese version of ECNQ-CCV is a reliable and valid scale with clinical utility in measuring ethical conflict. Health professionals can use it to measure the experience of exposure to ethical conflict in critical care nurses and attach great importance to the ramifications of these conflicts. Further evidence supporting its application is expected from a diverse population among Chinese critical care nurses.

## Supplementary Information

**Additional file 1: Supplementary 1.** Original Ethical Conflict Nursing Questionnaire - Critical Care Version. Supplementary 2: The Chinese version of the Ethical Conflict Nursing Questionnaire - Critical Care Version (ECNQ-CCV-C). Supplementary 3: Item analysis of the ECNQ-CCV-C (*n* = 248). Supplementary 4: Modified CFA of the ECNQ-CCV-C.

## Data Availability

The datasets used and analyzed during the current study are available from the corresponding author on reasonable request.

## Abbreviations

ECNQ-CCV: Ethical Conflict in Nursing Questionnaire-Critical Care Version
ECNQ-CCV-C: Chinese version of the Ethical Conflict in Nursing Questionnaire-Critical Care Version
ICU: Intensive care unit
MDS: Moral Distress Scale
MDS-R: Moral Distress Scale-Revision
HECS: Hospital Ethical Climate Survey
ProQOL: Professional Quality of Life Scale
COSMIN: Consensus-based standards for the selection of health status measurement instruments
STROBE: Strengthening the reporting of observational studies in epidemiology
EICU: Emergency intensive care unit
SICU: Surgical intensive care unit
CSICU: Cardiac surgical intensive care unit
NICU: Neurosurgery intensive care unit
SD: standard deviation
CFA: Confirmatory Factor Analysis models
SRMR: Standardized root mean square residual
RMSEA: Root mean square error of approximation
GFI: Goodness-of-fit index
CFI: Comparative fit index
MI: Modification indices
ICC: Intraclass correlation coefficients
SEM: Standard Error of Measurement
SDC: Smallest detectable change
