# Supplementary material for: Psychometric properties of the ethical conflict in nursing questionnaire critical care version among Chinese nurses: a cross-sectional study
Source: BMC Nurs. 2021 Jul 28;20:133. doi: 10.1186/s12912-021-00651-x (PMC8316889; doi:10.1186/s12912-021-00651-x)

Supplementary materials

Supplementary 1: Original Ethical Conflict Nursing Questionnaire - Critical Care Version

Care nursing scenarios	A. How often have you found yourself in this situation?	B. Has this situation been an ethical problem for you?	C. Please highlight the option that best describes the moral state that you have experienced in this situation.
**<u>Scenario 1</u>**<br>Administering treatments and/or performing tests that I consider unnecessary because they serve merely to prolong a terminal, irreversible process.	Never (Please do not answer B or C).		Moral indifference, because I not feel involved in this situation.
	Almost never.	Not a problem at all.	Moral wellbeing, because my moral thought and action are clearly coherent with one another.
	At least once a year.	Mildly problematic.	Moral uncertainty, because I'm not sure whether there is an ethical problem or not, or I recognize that there is a problem but is unclear about the ethical principles involved.
	At least once every six months.	Fairly problematic.	Moral dilemma, because I must choose between two or more morally correct principles, each of which would lead to a distinct course of action.
	At least once a month.	Considerably problematic.	Moral distress, because I recognize the ethical principles involved and I knows the right thing to do but is constrained by something or somebody from acting accordingly.
	At least once a week.	Highly problematic.	Moral outrage, because I experience a sense of impotence in the face of an immoral action performed by others.
**<u>Scenario 2</u>**<br>Having to administer treatments and/or carry out procedures without the critical patient, who is conscious, knowing their purpose and the risks involved.	Never (Please do not answer B or C).		Moral indifference, because I not feel involved in this situation.
	Almost never.	Not a problem at all.	Moral wellbeing, because my moral thought and action are clearly coherent with one another.
	At least once a year.	Mildly problematic.	Moral uncertainty, because I'm not sure whether there is an ethical problem or not, or I recognize that there is a problem but is unclear about the ethical principles involved.
	At least once every six months.	Fairly problematic.	Moral dilemma, because I must choose between two or more morally correct principles, each of which would lead to a distinct course of action.

	At least once a month.	Considerably problematic.	Moral distress, because I recognize the ethical principles involved and I knows the right thing to do but is constrained by something or somebody from acting accordingly.
	At least once a week.	Highly problematic.	Moral outrage, because I experience a sense of impotence in the face of an immoral action performed by others.
**Scenario 3** Caring for a patient who I believe should be on an ordinary hospital ward rather than in a critical care unit.	Never (Please do not answer B or C).		Moral indifference, because I not feel involved in this situation.
	Almost never.	Not a problem at all.	Moral wellbeing, because my moral thought and action are clearly coherent with one another.
	At least once a year.	Mildly problematic.	Moral uncertainty, because I'm not sure whether there is an ethical problem or not, or I recognize that there is a problem but is unclear about the ethical principles involved.
	At least once every six months.	Fairly problematic.	Moral dilemma, because I must choose between two or more morally correct principles, each of which would lead to a distinct course of action.
	At least once a month.	Considerably problematic.	Moral distress, because I recognize the ethical principles involved and I knows the right thing to do but is constrained by something or somebody from acting accordingly.
	At least once a week.	Highly problematic.	Moral outrage, because I experience a sense of impotence in the face of an immoral action performed by others.
**Scenario 4** Carrying out interventions that put institutional or health service interests before those of the patient.	Never (Please do not answer B or C).		Moral indifference, because I not feel involved in this situation.
	Almost never.	Not a problem at all.	Moral wellbeing, because my moral thought and action are clearly coherent with one another.
	At least once a year.	Mildly problematic.	Moral uncertainty, because I'm not sure whether there is an ethical problem or not, or I recognize that there is a problem but is unclear about the ethical principles involved.
	At least once every six months.	Fairly problematic.	Moral dilemma, because I must choose between two or more morally correct principles, each of which would lead to a distinct course of action.
	At least once a month.	Considerably problematic.	Moral distress, because I recognize the ethical principles involved and I knows the right thing to do but is constrained by something or somebody

			from acting accordingly.
	At least once a week.	Highly problematic.	Moral outrage, because I experience a sense of impotence in the face of an immoral action performed by others.
**Scenario 5**<br>Failure to keep a patient's clinical data confidential by sharing them with third parties or with people who are not directly involved in the patient's care.	Never (Please do not answer B or C).		Moral indifference, because I not feel involved in this situation.
	Almost never.	Not a problem at all.	Moral wellbeing, because my moral thought and action are clearly coherent with one another.
	At least once a year.	Mildly problematic.	Moral uncertainty, because I'm not sure whether there is an ethical problem or not, or I recognize that there is a problem but is unclear about the ethical principles involved.
	At least once every six months.	Fairly problematic.	Moral dilemma, because I must choose between two or more morally correct principles, each of which would lead to a distinct course of action.
	At least once a month.	Considerably problematic.	Moral distress, because I recognize the ethical principles involved and I knows the right thing to do but is constrained by something or somebody from acting accordingly.
	At least once a week.	Highly problematic.	Moral outrage, because I experience a sense of impotence in the face of an immoral action performed by others.
**Scenario 6**<br>Administering treatments and/or carrying out interventions without the patient's family knowing the objectives, benefits and risks involved (when the patient has consented to the family being informed)	Never (Please do not answer B or C).		Moral indifference, because I not feel involved in this situation.
	Almost never.	Not a problem at all.	Moral wellbeing, because my moral thought and action are clearly coherent with one another.
	At least once a year.	Mildly problematic.	Moral uncertainty, because I'm not sure whether there is an ethical problem or not, or I recognize that there is a problem but is unclear about the ethical principles involved.
	At least once every six months.	Fairly problematic.	Moral dilemma, because I must choose between two or more morally correct principles, each of which would lead to a distinct course of action.
	At least once a month.	Considerably problematic.	Moral distress, because I recognize the ethical principles involved and I knows the right thing to do but is constrained by something or somebody from acting accordingly.
	At least once a week.	Highly problematic.	Moral outrage, because I experience a sense of impotence in the face of an

			immoral action performed by others.
	Never (Please do not answer B or C).		Moral indifference, because I not feel involved in this situation.
	Almost never.	Not a problem at all.	Moral wellbeing, because my moral thought and action are clearly coherent with one another.
**Scenario 7**<br>Realizing that the analgesia and/or sedation being given to the patient is not effective enough and that the patient is suffering.	At least once a year.	Mildly problematic.	Moral uncertainty, because I'm not sure whether there is an ethical problem or not, or I recognize that there is a problem but is unclear about the ethical principles involved.
	At least once every six months.	Fairly problematic.	Moral dilemma, because I must choose between two or more morally correct principles, each of which would lead to a distinct course of action.
	At least once a month.	Considerably problematic.	Moral distress, because I recognize the ethical principles involved and I knows the right thing to do but is constrained by something or somebody from acting accordingly.
	At least once a week.	Highly problematic.	Moral outrage, because I experience a sense of impotence in the face of an immoral action performed by others.
	Never (Please do not answer B or C).		Moral indifference, because I not feel involved in this situation.
	Almost never.	Not a problem at all.	Moral wellbeing, because my moral thought and action are clearly coherent with one another.
**Scenario 8**<br>Using all available technical and/or human resources despite believing that they will produce no significant improvement in the clinical status of the critical care patient.	At least once a year.	Mildly problematic.	Moral uncertainty, because I'm not sure whether there is an ethical problem or not, or I recognize that there is a problem but is unclear about the ethical principles involved.
	At least once every six months.	Fairly problematic.	Moral dilemma, because I must choose between two or more morally correct principles, each of which would lead to a distinct course of action.
	At least once a month.	Considerably problematic.	Moral distress, because I recognize the ethical principles involved and I knows the right thing to do but is constrained by something or somebody from acting accordingly.
	At least once a week.	Highly problematic.	Moral outrage, because I experience a sense of impotence in the face of an immoral action performed by others.
**Scenario 9**	Never (Please do not answer B or C).		Moral indifference, because I not feel involved in this situation.

Working with medical staff who I consider to be professionally incompetent.	Almost never.	Not a problem at all.	Moral wellbeing, because my moral thought and action are clearly coherent with one another.
	At least once a year.	Mildly problematic.	Moral uncertainty, because I'm not sure whether there is an ethical problem or not, or I recognize that there is a problem but is unclear about the ethical principles involved.
	At least once every six months.	Fairly problematic.	Moral dilemma, because I must choose between two or more morally correct principles, each of which would lead to a distinct course of action.
	At least once a month.	Considerably problematic.	Moral distress, because I recognize the ethical principles involved and I knows the right thing to do but is constrained by something or somebody from acting accordingly.
	At least once a week.	Highly problematic.	Moral outrage, because I experience a sense of impotence in the face of an immoral action performed by others.
	Never (Please do not answer B or C).		Moral indifference, because I not feel involved in this situation.
	Almost never.	Not a problem at all.	Moral wellbeing, because my moral thought and action are clearly coherent with one another.
**<u>Scenario 10</u>**<br>Administering treatments and/or carrying out interventions in accordance with the family's wishes, despite knowing that these clash with the patient's interests.	At least once a year.	Mildly problematic.	Moral uncertainty, because I'm not sure whether there is an ethical problem or not, or I recognize that there is a problem but is unclear about the ethical principles involved.
	At least once every six months.	Fairly problematic.	Moral dilemma, because I must choose between two or more morally correct principles, each of which would lead to a distinct course of action.
	At least once a month.	Considerably problematic.	Moral distress, because I recognize the ethical principles involved and I knows the right thing to do but is constrained by something or somebody from acting accordingly.
	At least once a week.	Highly problematic.	Moral outrage, because I experience a sense of impotence in the face of an immoral action performed by others.
	Never (Please do not answer B or C).		Moral indifference, because I not feel involved in this situation.
**<u>Scenario 11</u>**	Almost never.	Not a problem at all.	Moral wellbeing, because my moral thought and action are clearly coherent with one another.

Administering treatments and/or carrying out procedures that are too aggressive given the status of the patient, and in so doing causing the patient additional suffering.	At least once a year.	Mildly problematic.	Moral uncertainty, because I'm not sure whether there is an ethical problem or not, or I recognize that there is a problem but is unclear about the ethical principles involved.
	At least once every six months.	Fairly problematic.	Moral dilemma, because I must choose between two or more morally correct principles, each of which would lead to a distinct course of action.
	At least once a month.	Considerably problematic.	Moral distress, because I recognize the ethical principles involved and I knows the right thing to do but is constrained by something or somebody from acting accordingly.
	At least once a week.	Highly problematic.	Moral outrage, because I experience a sense of impotence in the face of an immoral action performed by others.
**<u>Scenario 12</u>** Working with a nurse or nursing assistant who I consider to be professionally incompetent.	Never (Please do not answer B or C).		Moral indifference, because I not feel involved in this situation.
	Almost never.	Not a problem at all.	Moral wellbeing, because my moral thought and action are clearly coherent with one another.
	At least once a year.	Mildly problematic.	Moral uncertainty, because I'm not sure whether there is an ethical problem or not, or I recognize that there is a problem but is unclear about the ethical principles involved.
	At least once every six months.	Fairly problematic.	Moral dilemma, because I must choose between two or more morally correct principles, each of which would lead to a distinct course of action.
	At least once a month.	Considerably problematic.	Moral distress, because I recognize the ethical principles involved and I knows the right thing to do but is constrained by something or somebody from acting accordingly.
	At least once a week.	Highly problematic.	Moral outrage, because I experience a sense of impotence in the face of an immoral action performed by others.
**<u>Scenario 13</u>** Acting contrary to my own moral beliefs due to not having enough time to care properly for the patient.	Never (Please do not answer B or C).		Moral indifference, because I not feel involved in this situation.
	Almost never.	Not a problem at all.	Moral wellbeing, because my moral thought and action are clearly coherent with one another.
	At least once a year.	Mildly problematic.	Moral uncertainty, because I'm not sure whether there is an ethical problem or not, or I recognize that there is a problem but is unclear about

			the ethical principles involved.
	At least once every six months.	Fairly problematic.	Moral dilemma, because I must choose between two or more morally correct principles, each of which would lead to a distinct course of action.
	At least once a month.	Considerably problematic.	Moral distress, because I recognize the ethical principles involved and I knows the right thing to do but is constrained by something or somebody from acting accordingly.
	At least once a week.	Highly problematic.	Moral outrage, because I experience a sense of impotence in the face of an immoral action performed by others.
**<u>Scenario 14</u>** Administering treatments in the context of a clinical trial or research project without, as a nurse, being given all the information I consider necessary to carry out this task.	Never (Please do not answer B or C).		Moral indifference, because I not feel involved in this situation.
	Almost never.	Not a problem at all.	Moral wellbeing, because my moral thought and action are clearly coherent with one another.
	At least once a year.	Mildly problematic.	Moral uncertainty, because I'm not sure whether there is an ethical problem or not, or I recognize that there is a problem but is unclear about the ethical principles involved.
	At least once every six months.	Fairly problematic.	Moral dilemma, because I must choose between two or more morally correct principles, each of which would lead to a distinct course of action.
	At least once a month.	Considerably problematic.	Moral distress, because I recognize the ethical principles involved and I knows the right thing to do but is constrained by something or somebody from acting accordingly.
	At least once a week.	Highly problematic.	Moral outrage, because I experience a sense of impotence in the face of an immoral action performed by others.
**<u>Scenario 15</u>** Finding it difficult to give timely information to the patient and/or his/her family because the medical team	Never (Please do not answer B or C).		Moral indifference, because I not feel involved in this situation.
	Almost never.	Not a problem at all.	Moral wellbeing, because my moral thought and action are clearly coherent with one another.
	At least once a year.	Mildly problematic.	Moral uncertainty, because I'm not sure whether there is an ethical problem or not, or I recognize that there is a problem but is unclear about the ethical principles involved.

discourages nurses from taking the initiative in this regard.	At least once every six months.	Fairly problematic.	Moral dilemma, because I must choose between two or more morally correct principles, each of which would lead to a distinct course of action.
	At least once a month.	Considerably problematic.	Moral distress, because I recognize the ethical principles involved and I knows the right thing to do but is constrained by something or somebody from acting accordingly.
	At least once a week.	Highly problematic.	Moral outrage, because I experience a sense of impotence in the face of an immoral action performed by others.
**Scenario 16**<br>Caring for a patient without knowing whether or not he or she has made a living will declaration, or in the event that such a document exists not knowing its content.	Never (Please do not answer B or C).		Moral indifference, because I not feel involved in this situation.
	Almost never.	Not a problem at all.	Moral wellbeing, because my moral thought and action are clearly coherent with one another.
	At least once a year.	Mildly problematic.	Moral uncertainty, because I'm not sure whether there is an ethical problem or not, or I recognize that there is a problem but is unclear about the ethical principles involved.
	At least once every six months.	Fairly problematic.	Moral dilemma, because I must choose between two or more morally correct principles, each of which would lead to a distinct course of action.
	At least once a month.	Considerably problematic.	Moral distress, because I recognize the ethical principles involved and I knows the right thing to do but is constrained by something or somebody from acting accordingly.
	At least once a week.	Highly problematic.	Moral outrage, because I experience a sense of impotence in the face of an immoral action performed by others.
**Scenario 17**<br>Administering treatments and/or carrying out procedures without, as a nurse, having been previously involved in the decision to do so.	Never (Please do not answer B or C).		Moral indifference, because I not feel involved in this situation.
	Almost never.	Not a problem at all.	Moral wellbeing, because my moral thought and action are clearly coherent with one another.
	At least once a year.	Mildly problematic.	Moral uncertainty, because I'm not sure whether there is an ethical problem or not, or I recognize that there is a problem but is unclear about the ethical principles involved.
	At least once every six months.	Fairly problematic.	Moral dilemma, because I must choose between two or more morally correct principles, each of which would lead to a distinct course of action.

	At least once a month.	Considerably problematic.	Moral distress, because I recognize the ethical principles involved and I knows the right thing to do but is constrained by something or somebody from acting accordingly.
	At least once a week.	Highly problematic.	Moral outrage, because I experience a sense of impotence in the face of an immoral action performed by others.
**<u>Scenario 18</u>** Failure to respect properly the privacy of the patient's body when carrying out procedures and/or exploratory tests.	Never (Please do not answer B or C).		Moral indifference, because I not feel involved in this situation.
	Almost never.	Not a problem at all.	Moral wellbeing, because my moral thought and action are clearly coherent with one another.
	At least once a year.	Mildly problematic.	Moral uncertainty, because I'm not sure whether there is an ethical problem or not, or I recognize that there is a problem but is unclear about the ethical principles involved.
	At least once every six months.	Fairly problematic.	Moral dilemma, because I must choose between two or more morally correct
			principles, each of which would lead to a distinct course of action.
	At least once a month.	Considerably problematic.	Moral distress, because I recognize the ethical principles involved and I knows the right thing to do but is constrained by something or somebody from acting accordingly.
	At least once a week.	Highly problematic.	Moral outrage, because I experience a sense of impotence in the face of an immoral action performed by others.
**<u>Scenario 19</u>** Lacking the means (space) and/or resources (time) that would enable the clinical team to consider the ethical problems they have to face.	Never (Please do not answer B or C).		Moral indifference, because I not feel involved in this situation.
	Almost never.	Not a problem at all.	Moral wellbeing, because my moral thought and action are clearly coherent with one another.
	At least once a year.	Mildly problematic.	Moral uncertainty, because I'm not sure whether there is an ethical problem or not, or I recognize that there is a problem but is unclear about the ethical principles involved.
	At least once every six months.	Fairly problematic.	Moral dilemma, because I must choose between two or more morally correct principles, each of which would lead to a distinct course of action.

	At least once a month.	Considerably problematic.	Moral distress, because I recognize the ethical principles involved and I knows the right thing to do but is constrained by something or somebody from acting accordingly.
	At least once a week.	Highly problematic.	Moral outrage, because I experience a sense of impotence in the face of an immoral action performed by others.

(*) The analysis of the psychometric properties of the ECNQ was carried out in relation to the original Spanish version of the instrument.

(**) You may request permission to author to use the questionnaire.

Types of ethical conflicts in ECNQ-CCV

Moral indifference describes the stance of an individual who neither shows interest in nor takes a position on a matter of ethical concern.
Moral wellbeing describes to a positive state in which moral thought and action are clearly coherent with one another.
Moral uncertainty describes the states of an individual who either unsure whether there is an ethical problem or not, or recognizes that there is such a problem but is unclear about the ethical principles involved.
Moral dilemma arise when the individual must choose between two or more morally correct principles, each of which would lead to a distinct course of action.
Moral distress is felt when the individual recognizes the ethical principles involved and knows the right thing to do but is constrained by something or somebody from acting accordingly.
Moral outrage is a type of ethical conflict in which the individual experiences a sense of impotence in face of an immoral action performed by others.

Relations between conflict areas and scenarios of ECNQ-CCV

Informed consent: Scenario 2, Scenario 6.
Confidentiality: Scenario 5.
Withholding and withdrawing treatments: Scenario 1.
Patient’s interests: Scenario 4, Scenario 10; Scenario 16.
Characteristics of an ethical environment: Scenario 19.
Procedures and treatments: Scenario 7, Scenario 11.
Interprofessional relationships: Scenario 9, Scenario 12, Scenario 15, Scenario 17.
Moral agency and professional values: Scenario 13.
Privacy: Scenario 18.
Research tasks: Scenario 14.
Resource management: Scenario 3, Scenario 8.

Supplementary 2: The Chinese version of the Ethical Conflict Nursing Questionnaire - Critical Care Version (ECNQ-CCV-C)

中文版重症监护护理伦理冲突问卷

护理情景	A 你多久遇到一次这种情况？	B 这种情景对你来说是一个伦理问题吗？	C 请选出跟你在这种情景下的道德感受及认知最符合的选项
情景 1<br>我执行了我认为是不必要的治疗和/或检查，因为它们只能延长患者临终的、不可逆转的阶段	从来没有（请不用回答 B、C）<br>几乎没有<br>至少每年一次<br>至少每六个月一次<br>至少每月一次<br>至少每周一次	根本没有这个问题<br>是一个小问题<br>是一个中等程度的问题<br>是一个大问题<br>是一个严重的问题	道德冷漠，因为我感觉自己没有卷入这种情况<br>道德幸福，因为我在道德上的想法和行为非常一致<br>道德不确定，因为我不确定这是不是一个伦理问题；或者我意识到这是个伦理问题，但说不出来这涉及到的伦理原则是什么<br>道德两难，因为我必须在两个或以上道德上正确的原则之间做出选择，而每一个选择都会导致不同的行动方案<br>道德困境，因为我意识到其中涉及的伦理原则，并且我知道怎么做是对的，但受制于某些事情或某些人，我无法采取相应的行动<br>道德义愤，因为我在面对别人的不道德行为时感到无能为力
情景 2<br>我不得不对意识清楚的重症患者执行一些治疗和/或操作，虽然他们并不知道这些治疗和/或操作的目的和风险	从来没有（请不用回答 B、C）<br>几乎没有<br>至少每年一次<br>至少每六个月一次<br>至少每月一次<br>至少每周一次	根本没有这个问题<br>是一个小问题<br>是一个中等程度的问题<br>是一个大问题<br>是一个严重的问题	道德冷漠，因为我感觉自己没有卷入这种情况<br>道德幸福，因为我在道德上的想法和行为非常一致<br>道德不确定，因为我不确定这是不是一个伦理问题；或者我意识到这是个伦理问题，但说不出来这涉及到的伦理原则是什么<br>道德两难，因为我必须在两个或以上道德上正确的原则之间做出选择，而每一个选择都会导致不同的行动方案<br>道德困境，因为我意识到其中涉及的伦理原则，并且我知道怎么做是对的，但受制于某些事情或某些人，我无法采取相应的行动<br>道德义愤，因为我在面对别人的不道德行为时感到无能为力

情景 3<br>我照顾了一个我认为本应该在普通病房而不是重症监护室的患者	从来没有（请不用回答 B、C）<br>几乎没有<br>至少每年一次<br>至少每六个月一次<br>至少每月一次<br>至少每周一次	根本没有这个问题<br>是一个小问题<br>是一个中等程度的问题<br>是一个大问题<br>是一个严重的问题	道德冷漠，因为我感觉自己没有卷入这种情况<br>道德幸福，因为我在道德上的想法和行为非常一致<br>道德不确定，因为我不确定这是不是一个伦理问题；或者我意识到这是个伦理问题，但说不出来这涉及到的伦理原则是什么<br>道德两难，因为我必须在两个或以上道德上正确的原则之间做出选择，而每一个选择都会导致不同的行动方案<br>道德困境，因为我意识到其中涉及的伦理原则，并且我知道怎么做是对的，但受制于某些事情或某些人，我无法采取相应的行动<br>道德义愤，因为我在面对别人的不道德行为时感到无能为力力
情景 4<br>我执行的操作首先考虑的是机构或卫生服务的利益，而不是患者的利益	从来没有（请不用回答 B、C）<br>几乎没有<br>至少每年一次<br>至少每六个月一次<br>至少每月一次<br>至少每周一次	根本没有这个问题<br>是一个小问题<br>是一个中等程度的问题<br>是一个大问题<br>是一个严重的问题	道德冷漠，因为我感觉自己没有卷入这种情况<br>道德幸福，因为我在道德上的想法和行为非常一致<br>道德不确定，因为我不确定这是不是一个伦理问题；或者我意识到这是个伦理问题，但说不出来这涉及到的伦理原则是什么<br>道德两难，因为我必须在两个或以上道德上正确的原则之间做出选择，而每一个选择都会导致不同的行动方案<br>道德困境，因为我意识到其中涉及的伦理原则，并且我知道怎么做是对的，但受制于某些事情或某些人，我无法采取相应的行动<br>道德义愤，因为我在面对别人的不道德行为时感到无能为力

情景 5<br>我没能保护好患者的住院信息，将其与第三方或没有直接参与患者照护的人分享	从来没有（请不用回答 B、C）<br>几乎没有<br>至少每年一次<br>至少每六个月一次<br>至少每月一次<br>至少每周一次	根本没有这个问题<br>是一个小问题<br>是一个中等程度的问题<br>是一个大问题<br>是一个严重的问题	道德冷漠，因为我感觉自己没有卷入这种情况<br>道德幸福，因为我在道德上的想法和行为非常一致<br>道德不确定，因为我不确定这是不是一个伦理问题；或者我意识到这是个伦理问题，但说不出来这涉及到的伦理原则是什么<br>道德两难，因为我必须在两个或以上道德上正确的原则之间做出选择，而每一个选择都会导致不同的行动方案<br>道德困境，因为我意识到其中涉及的伦理原则，并且我知道怎么做是对的，但受制于某些事情或某些人，我无法采取相应的行动<br>道德义愤，因为我在面对别人的不道德行为时感到无能为力
情景 6<br>我执行的治疗和/或操作并未让家属知晓治疗目的、益处以及风险（虽然患者同意家属有知情权）	从来没有（请不用回答 B、C）<br>几乎没有<br>至少每年一次<br>至少每六个月一次<br>至少每月一次<br>至少每周一次	根本没有这个问题<br>是一个小问题<br>是一个中等程度的问题<br>是一个大问题<br>是一个严重的问题	道德冷漠，因为我感觉自己没有卷入这种情况<br>道德幸福，因为我在道德上的想法和行为非常一致<br>道德不确定，因为我不确定这是不是一个伦理问题；或者我意识到这是个伦理问题，但说不出来这涉及到的伦理原则是什么<br>道德两难，因为我必须在两个或以上道德上正确的原则之间做出选择，而每一个选择都会导致不同的行动方案<br>道德困境，因为我意识到其中涉及的伦理原则，并且我知道怎么做是对的，但受制于某些事情或某些人，我无法采取相应的行动<br>道德义愤，因为我在面对别人的不道德行为时感到无能为力

情景 7<br>我意识到给患者用的镇痛和/或镇静药并没有足够的疗效，患者依然很痛苦	从来没有（请不用回答 B、C）<br>几乎没有<br>至少每年一次<br>至少每六个月一次<br>至少每月一次<br>至少每周一次	根本没有这个问题<br>是一个小问题<br>是一个中等程度的问题<br>是一个大问题<br>是一个严重的问题	道德冷漠，因为我感觉自己没有卷入这种情况<br>道德幸福，因为我在道德上的想法和行为非常一致<br>道德不确定，因为我不确定这是不是一个伦理问题；或者我意识到这是个伦理问题，但说不出来这涉及到的伦理原则是什么<br>道德两难，因为我必须在两个或以上道德上正确的原则之间做出选择，而每一个选择都会导致不同的行动方案<br>道德困境，因为我意识到其中涉及的伦理原则，并且我知道怎么做是对的，但受制于某些事情或某些人，我无法采取相应的行动<br>道德义愤，因为我在面对别人的不道德行为时感到无能为力
情景 8<br>我对危重患者倾尽可用的技术和/或人力资源，尽管我清楚这样做并不能显著改善危重患者的临床结局	从来没有（请不用回答 B、C）<br>几乎没有<br>至少每年一次<br>至少每六个月一次<br>至少每月一次<br>至少每周一次	根本没有这个问题<br>是一个小问题<br>是一个中等程度的问题<br>是一个大问题<br>是一个严重的问题	道德冷漠，因为我感觉自己没有卷入这种情况<br>道德幸福，因为我在道德上的想法和行为非常一致<br>道德不确定，因为我不确定这是不是一个伦理问题；或者我意识到这是个伦理问题，但说不出来这涉及到的伦理原则是什么<br>道德两难，因为我必须在两个或以上道德上正确的原则之间做出选择，而每一个选择都会导致不同的行动方案<br>道德困境，因为我意识到其中涉及的伦理原则，并且我知道怎么做是对的，但受制于某些事情或某些人，我无法采取相应的行动<br>道德义愤，因为我在面对别人的不道德行为时感到无能为力

情景 9<br>我和我认为岗位胜任力差的医生一起工作	从来没有（请不用回答 B、C）<br>几乎没有<br>至少每年一次<br>至少每六个月一次<br>至少每月一次<br>至少每周一次	根本没有这个问题<br>是一个小问题<br>是一个中等程度的问题<br>是一个大问题<br>是一个严重的问题	道德冷漠，因为我感觉自己没有卷入这种情况<br>道德幸福，因为我在道德上的想法和行为非常一致<br>道德不确定，因为我不确定这是不是一个伦理问题；或者我意识到这是个伦理问题，但说不出来这涉及到的伦理原则是什么<br>道德两难，因为我必须在两个或以上道德上正确的原则之间做出选择，而每一个选择都会导致不同的行动方案<br>道德困境，因为我意识到其中涉及的伦理原则，并且我知道怎么做是对的，但受制于某些事情或某些人，我无法采取相应的行动<br>道德义愤，因为我在面对别人的不道德行为时感到无能为力
情景 10<br>我根据家属的意愿执行治疗和/或操作，尽管知道这与患者利益冲突	从来没有（请不用回答 B、C）<br>几乎没有<br>至少每年一次<br>至少每六个月一次<br>至少每月一次<br>至少每周一次	根本没有这个问题<br>是一个小问题<br>是一个中等程度的问题<br>是一个大问题<br>是一个严重的问题	道德冷漠，因为我感觉自己没有卷入这种情况<br>道德幸福，因为我在道德上的想法和行为非常一致<br>道德不确定，因为我不确定这是不是一个伦理问题；或者我意识到这是个伦理问题，但说不出来这涉及到的伦理原则是什么<br>道德两难，因为我必须在两个或以上道德上正确的原则之间做出选择，而每一个选择都会导致不同的行动方案<br>道德困境，因为我意识到其中涉及的伦理原则，并且我知道怎么做是对的，但受制于某些事情或某些人，我无法采取相应的行动<br>道德义愤，因为我在面对别人的不道德行为时感到无能为力

情景 11<br>我对患者执行过度的治疗和/或操作，并因此给患者带来额外的痛苦	从来没有（请不用回答 B、C）<br>几乎没有<br>至少每年一次<br>至少每六个月一次<br>至少每月一次<br>至少每周一次	根本没有这个问题<br>是一个小问题<br>是一个中等程度的问题<br>是一个大问题<br>是一个严重的问题	道德冷漠，因为我感觉自己没有卷入这种情况<br>道德幸福，因为我在道德上的想法和行为非常一致<br>道德不确定，因为我不确定这是不是一个伦理问题；或者我意识到这是个伦理问题，但说不出来这涉及到的伦理原则是什么<br>道德两难，因为我必须在两个或以上道德上正确的原则之间做出选择，而每一个选择都会导致不同的行动方案<br>道德困境，因为我意识到其中涉及的伦理原则，并且我知道怎么做是对的，但受制于某些事情或某些人，我无法采取相应的行动<br>道德义愤，因为我在面对别人的不道德行为时感到无能为力
情景 12<br>我和我认为岗位胜任力差的护士或助理护士工作	从来没有（请不用回答 B、C）<br>几乎没有<br>至少每年一次<br>至少每六个月一次<br>至少每月一次<br>至少每周一次	根本没有这个问题<br>是一个小问题<br>是一个中等程度的问题<br>是一个大问题<br>是一个严重的问题	道德冷漠，因为我感觉自己没有卷入这种情况<br>道德幸福，因为我在道德上的想法和行为非常一致<br>道德不确定，因为我不确定这是不是一个伦理问题；或者我意识到这是个伦理问题，但说不出来这涉及到的伦理原则是什么<br>道德两难，因为我必须在两个或以上道德上正确的原则之间做出选择，而每一个选择都会导致不同的行动方案<br>道德困境，因为我意识到其中涉及的伦理原则，并且我知道怎么做是对的，但受制于某些事情或某些人，我无法采取相应的行动<br>道德义愤，因为我在面对别人的不道德行为时感到无能为力

情景 13<br>我采取了违背自己道德信仰的行为，是因为没有足够的时间去规范地照顾患者	从来没有（请不用回答 B、C）<br>几乎没有<br>至少每年一次<br>至少每六个月一次<br>至少每月一次<br>至少每周一次	根本没有这个问题<br>是一个小问题<br>是一个中等程度的问题<br>是一个大问题<br>是一个严重的问题	道德冷漠，因为我感觉自己没有卷入这种情况<br>道德幸福，因为我在道德上的想法和行为非常一致<br>道德不确定，因为我不确定这是不是一个伦理问题；或者我意识到这是个伦理问题，但说不出来这涉及到的伦理原则是什么<br>道德两难，因为我必须在两个或以上道德上正确的原则之间做出选择，而每一个选择都会导致不同的行动方案<br>道德困境，因为我意识到其中涉及的伦理原则，并且我知道怎么做是对的，但受制于某些事情或某些人，我无法采取相应的行动<br>道德义愤，因为我在面对别人的不道德行为时感到无能为力
情景 14<br>我作为护士，在一个临床试验或研究中，没有获得我认为需要的所有信息就去执行医嘱	从来没有（请不用回答 B、C）<br>几乎没有<br>至少每年一次<br>至少每六个月一次<br>至少每月一次<br>至少每周一次	根本没有这个问题<br>是一个小问题<br>是一个中等程度的问题<br>是一个大问题<br>是一个严重的问题	道德冷漠，因为我感觉自己没有卷入这种情况<br>道德幸福，因为我在道德上的想法和行为非常一致<br>道德不确定，因为我不确定这是不是一个伦理问题；或者我意识到这是个伦理问题，但说不出来这涉及到的伦理原则是什么<br>道德两难，因为我必须在两个或以上道德上正确的原则之间做出选择，而每一个选择都会导致不同的行动方案<br>道德困境，因为我意识到其中涉及的伦理原则，并且我知道怎么做是对的，但受制于某些事情或某些人，我无法采取相应的行动<br>道德义愤，因为我在面对别人的不道德行为时感到无能为力

情景 15<br>我发现很难及时向患者和/或家属提供信息，因为医疗团队没有鼓励护士主动提供相关信息	从来没有（请不用回答 B、C）<br>几乎没有<br>至少每年一次<br>至少每六个月一次<br>至少每月一次<br>至少每周一次	根本没有这个问题<br>是一个小问题<br>是一个中等程度的问题<br>是一个大问题<br>是一个严重的问题	道德冷漠，因为我感觉自己没有卷入这种情况<br>道德幸福，因为我在道德上的想法和行为非常一致<br>道德不确定，因为我不确定这是不是一个伦理问题；或者我意识到这是个伦理问题，但说不出来这涉及到的伦理原则是什么<br>道德两难，因为我必须在两个或以上道德上正确的原则之间做出选择，而每一个选择都会导致不同的行动方案<br>道德困境，因为我意识到其中涉及的伦理原则，并且我知道怎么做是对的，但受制于某些事情或某些人，我无法采取相应的行动<br>道德义愤，因为我在面对别人的不道德行为时感到无能为力
情景 16<br>我不知道我照护的患者是否有生前遗嘱，或者我知道他/她有生前遗嘱但不知道具体内容	从来没有（请不用回答 B、C）<br>几乎没有<br>至少每年一次<br>至少每六个月一次<br>至少每月一次<br>至少每周一次	根本没有这个问题<br>是一个小问题<br>是一个中等程度的问题<br>是一个大问题<br>是一个严重的问题	道德冷漠，因为我感觉自己没有卷入这种情况<br>道德幸福，因为我在道德上的想法和行为非常一致<br>道德不确定，因为我不确定这是不是一个伦理问题；或者我意识到这是个伦理问题，但说不出来这涉及到的伦理原则是什么<br>道德两难，因为我必须在两个或以上道德上正确的原则之间做出选择，而每一个选择都会导致不同的行动方案<br>道德困境，因为我意识到其中涉及的伦理原则，并且我知道怎么做是对的，但受制于某些事情或某些人，我无法采取相应的行动<br>道德义愤，因为我在面对别人的不道德行为时感到无能为力

情景 17<br>我作为一名护士，在没有事先参与决策的情况下就执行了治疗和/或操作	从来没有（请不用回答 B、C）<br>几乎没有<br>至少每年一次<br>至少每六个月一次<br>至少每月一次<br>至少每周一次	根本没有这个问题<br>是一个小问题<br>是一个中等程度的问题<br>是一个大问题<br>是一个严重的问题	道德冷漠，因为我感觉自己没有卷入这种情况<br>道德幸福，因为我在道德上的想法和行为非常一致<br>道德不确定，因为我不确定这是不是一个伦理问题；或者我意识到这是个伦理问题，但说不出来这涉及到的伦理原则是什么<br>道德两难，因为我必须在两个或以上道德上正确的原则之间做出选择，而每一个选择都会导致不同的行动方案<br>道德困境，因为我意识到其中涉及的伦理原则，并且我知道怎么做是对的，但受制于某些事情或某些人，我无法采取相应的行动<br>道德义愤，因为我在面对别人的不道德行为时感到无能为力
情景 18<br>我在执行操作和/或进行身体评估时，没能尊重患者的隐私	从来没有（请不用回答 B、C）<br>几乎没有<br>至少每年一次<br>至少每六个月一次<br>至少每月一次<br>至少每周一次	根本没有这个问题<br>是一个小问题<br>是一个中等程度的问题<br>是一个大问题<br>是一个严重的问题	道德冷漠，因为我感觉自己没有卷入这种情况<br>道德幸福，因为我在道德上的想法和行为非常一致<br>道德不确定，因为我不确定这是不是一个伦理问题；或者我意识到这是个伦理问题，但说不出来这涉及到的伦理原则是什么<br>道德两难，因为我必须在两个或以上道德上正确的原则之间做出选择，而每一个选择都会导致不同的行动方案<br>道德困境，因为我意识到其中涉及的伦理原则，并且我知道怎么做是对的，但受制于某些事情或某些人，我无法采取相应的行动<br>道德义愤，因为我在面对别人的不道德行为时感到无能为力

情景 19<br>环境中缺乏必要的途径（如空间）和/或资源（如时间），促使临床团队考虑他们必须面对的伦理问题	从来没有（请不用回答 B、C）<br>几乎没有<br>至少每年一次<br>至少每六个月一次<br>至少每月一次<br>至少每周一次	根本没有这个问题<br>是一个小问题<br>是一个中等程度的问题<br>是一个大问题<br>是一个严重的问题	道德冷漠，因为我感觉自己没有卷入这种情况<br>道德幸福，因为我在道德上的想法和行为非常一致<br>道德不确定，因为我不确定这是不是一个伦理问题；或者我意识到这是个伦理问题，但说不出来这涉及到的伦理原则是什么<br>道德两难，因为我必须在两个或以上道德上正确的原则之间做出选择，而每一个选择都会导致不同的行动方案<br>道德困境，因为我意识到其中涉及的伦理原则，并且我知道怎么做是对的，但受制于某些事情或某些人，我无法采取相应的行动<br>道德义愤，因为我在面对别人的不道德行为时感到无能为力

（*）对 ECNQ 的心理测量特性的分析是根据与西班牙语原始版本的对比得出的

（**）您可以向作者提出申请去使用这份量表

ECNQ-CCV 中的伦理冲突类型

道德冷漠：个人对伦理问题既不表示出兴趣也不表达立场的态度

道德幸福：个人道德上的想法和行为非常一致的积极状态

道德不确定：个人不确定是不是一个伦理问题；或者意识到是个伦理问题，但说不出来涉及到的伦理原则是什么

道德两难：个人必须在两个或以上道德上正确的原则之间做出选择，而每一个选择都会导致不同的行动方案

道德困境：个人意识到其中涉及的伦理原则，并且知道怎么做是对的，但受制于某些事情或某些人，无法采取相应的行动

道德义愤：个人在面对别人的不道德行为时感到无能为力，是一种伦理冲突的体现

冲突领域和 ECNQ-CCV 中情景的对应关系

知情同意原则：情景 2、6

保密原则：情景 5

维持和停止治疗原则：情景 1

患者利益原则：情景 4、10、16

伦理环境特征原则：情景 19

操作和治疗原则：情景 7、11

跨专业关系原则：情景 9、12、15、17

道德能力和专业价值观原则：情景 13

隐私原则：情景 18

研究任务原则：情景 14

资源管理原则：情景 3、8

Supplementary 3: Item analysis of the ECNQ-CCV-C (n=248)

ECNQ-CCV-C items	Adjusted item total correlation	Cronbach's α if the item is deleted	McDonald's ω if the item is deleted	Standerdized factor loading
Q1	0.410	0.900	0.902	0.425*
Q2	0.478	0.899	0.900	0.495*
Q3	0.436	0.900	0.901	0.452*
Q4	0.527	0.897	0.899	0.549*
Q5	0.488	0.898	0.900	0.513*
Q6	0.480	0.899	0.900	0.513*
Q7	0.549	0.897	0.898	0.568*
Q8	0.567	0.896	0.898	0.580*
Q9	0.545	0.897	0.898	0.577*
Q10	0.640	0.894	0.896	0.676*
Q11	0.664	0.893	0.895	0.704*
Q12	0.578	0.896	0.897	0.609*
Q13	0.599	0.895	0.897	0.642*
Q14	0.588	0.896	0.897	0.619*
Q15	0.590	0.896	0.897	0.625*
Q16	0.446	0.900	0.901	0.491*
Q17	0.609	0.895	0.896	0.648*
Q18	0.566	0.896	0.898	0.601*
Q19	0.544	0.897	0.898	0.581*

*$P < 0.01$; The Cronbach's α = 0.902, McDonald's ω = 0.903, Guttman Split-Half coefficient = 0.920, Spearman-Brown coefficient = 0.925

Supplementary 4: Modified CFA of the ECNQ-CCV-C

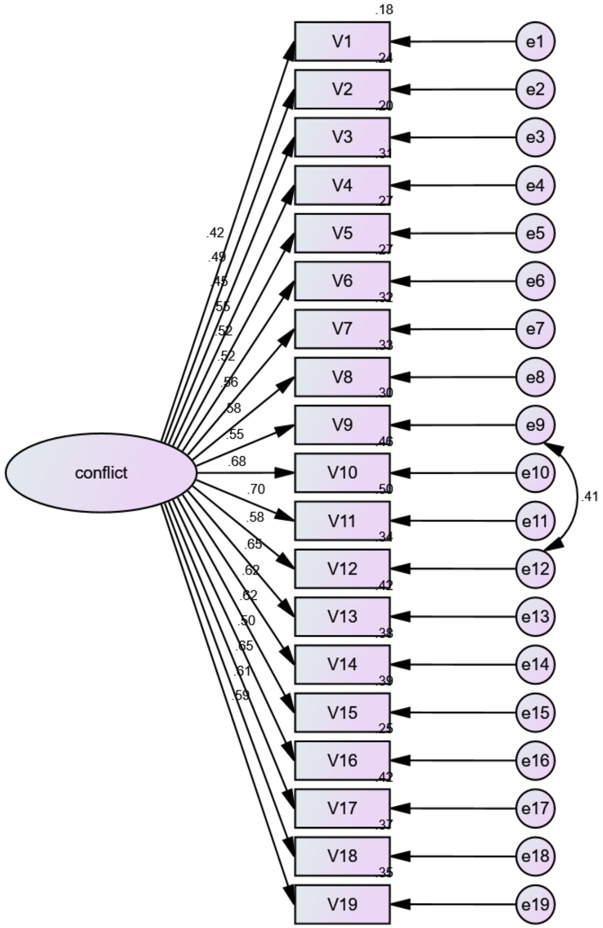

Supplement: Supplementary file 1 — Additional file 1: Supplementary 1. Original Ethical Conflict Nursing Questionnaire - Critical Care Version. Supplementary 2: The Chinese version of the Ethical Conflict Nursing Questionnaire - Critical Care Version (ECNQ-CCV-C). Supplementary 3: Item analysis of the ECNQ-CCV-C (n = 248). Supplementary 4: Modified CFA of the ECNQ-CCV-C. [file 12912_2021_651_MOESM1_ESM.pdf]
